# New Membrane Active Antibacterial and Antiviral Amphiphiles Derived from Heterocyclic Backbone of Pyridinium-4-Aldoxime

**DOI:** 10.3390/ph15070775

**Published:** 2022-06-22

**Authors:** Doris Crnčević, Lucija Krce, Mislav Cvitković, Zlatko Brkljača, Antonio Sabljić, Elma Vuko, Ines Primožič, Renata Odžak, Matilda Šprung

**Affiliations:** 1 Department of Chemistry, Faculty of Science, University of Split, R. Bošković 33, 21 000 Split, Croatia; dcrncevic@pmfst.hr (D.C.); asabljic1@pmfst.hr (A.S.); 2 Doctoral Study of Biophysics, Faculty of Science, University of Split, R. Bošković 33, 21 000 Split, Croatia; 3 Department of Physics, Faculty of Science, University of Split, R. Bošković 33, 21 000 Split, Croatia; lucija.krce@pmfst.hr (L.K.); mcvitkovi@pmfst.hr (M.C.); 4 Division of Organic Chemistry and Biochemistry, Ruđer Bošković Institute, Bijenička c. 54, 10 000 Zagreb, Croatia; zlatko.brkljaca@irb.hr; 5 Selvita Ltd., Prilaz Baruna Filipovića 29, 10 000 Zagreb, Croatia; 6 Department of Biology, Faculty of Science, University of Split, R. Bošković 33, 21 000 Split, Croatia; elma@pmfst.hr; 7 Department of Chemistry, Faculty of Science, University of Zagreb, Horvatovac 102a, 10 000 Zagreb, Croatia; ines.primozic@chem.pmf.hr

**Keywords:** quaternary ammonium salts, pyridinium-4-aldoxime, antimicrobial activity, cytotoxicity, mode of action mechanism

## Abstract

Quaternary ammonium salts (QAS) are irreplaceable membrane-active antimicrobial agents that have been widely used for nearly a century. Cetylpyridinium chloride (CPC) is one of the most potent QAS. However, recent data from the literature indicate that CPC activity against resistant bacterial strains is decreasing. The major QAS resistance pathway involves the QacR dimer, which regulates efflux pump expression. A plausible approach to address this issue is to structurally modify the CPC structure by adding other biologically active functional groups. Here, a series of QAS based on pyridine-4-aldoxime were synthesized, characterized, and tested for antimicrobial activity in vitro. Although we obtained several potent antiviral candidates, these candidates had lower antibacterial activity than CPC and were not toxic to human cell lines. We found that the addition of an oxime group to the pyridine backbone resulted in derivatives with large topological polar surfaces and with unfavorable cLog *P* values. Investigation of the antibacterial mode of action, involving the cell membrane, revealed altered cell morphologies in terms of corrugated and/or disrupted surface, while 87% of the cells studied exhibited a permeabilized membrane after 3 h of treatment at 4 × minimum inhibitory concentration (MIC). Molecular dynamic (MD) simulations of the interaction of QacR with a representative candidate showed rapid dimer disruption, whereas this was not observed for QacR and QacR bound to the structural analog CPC. This might explain the lower bioactivity of our compounds, as they are likely to cause premature expression of efflux pumps and thus activation of resistance.

## 1. Introduction

Antimicrobial membrane-active amphiphiles, such as quaternary ammonium salts (QAS), are important antiseptic and disinfectant agents in many different commercial products. QAS with permanent positive charge can occur naturally as a product of microbial secondary metabolism or can be synthesized from alkyl halides, alcohols, or amides [1,2]. The first QAS introduced to the disinfectant market in the 1930s and 1940s was benzalkonium chloride (BAC), and today there are other common QAS besides this, namely bromine and chlorine salts such as benzalkonium bromide (BAB), cetylpyridinium chloride (CPC), and didecyldimethylammonium chloride (DDAC) [3]. Due to the amphiphilic nature of QAS, they exhibit a detergent-like mode of action that targets the cell membrane. This involves electrostatic interaction between the negatively charged bacterial membrane and the positive charge of the QAS, whereupon the alkyl chain penetrates the lipid bilayer, leading to the release of the intracellular contents and cell death [3,4,5].

Although these agents are already widely used, some projections indicate that their consumption will increase in the coming years as public health is threatened by the current and future spread of infectious diseases [6]. Due to their increased chemical stability and longer retention time in the environment, there are legitimate concerns about the emergence of bacterial resistance to QAS. In 1999, 63% of methicillin-resistant *Staphylococcus aureus* (MRSA) isolates in Europe were already resistant to this class of compounds [7] and in 2012, 83% were resistant in the United States [8]. Commercial QAS can have 2- to 4-fold lower efficacy against resistant bacteria, particularly CPC, one of the most effective commercial disinfectants, which has 4-fold lower efficacy against MRSA in the biofilm state [1]. It was found that the major acquired resistance pathway in Gram-positive bacteria is the *qacA*/*qacR* system [3]. This system consists of the negative transcriptional regulator QacR, which binds to the IR1 site of DNA, thereby inactivating transcription of the QacA efflux pump [9]. QacR ligands are various aryl-substituted mono- and biscationic QAS that, when bound to QacR, induce a conformational change leading to its dissociation from DNA and production of the QacA efflux pump [10,11]. Therefore, solving the problem of bacterial resistance by developing new or structurally modified QAS is the focus of further scientific investigation.

Several research groups have made considerable efforts to develop new QAS with structural modifications of CPC [1,2,12,13,14,15]. In this sense, environmentally friendly CPC derivatives (“soft QAS”) have been synthesized [1]. Although these pyridinium derivatives have the potential to decompose more rapidly, which could halt the development of bacterial resistance, they have been less effective because antimicrobial activity has been shown to be closely related to the structural stability of these compounds. On the other hand, pyridinium derivatives with more than one positive center and different alkyl chain lengths have shown potent antimicrobial activity at low micromolar concentrations, most likely due to a multicationic-specific interaction with the negatively charged bacterial surface [12,13,14,16]. In addition, the authors speculate that QAS bearing more than two positive centers may be poor substrates for QacR, thus repressing the expression of specific efflux pumps.

QSAR studies have shown that various properties such as substituents on the pyridine moiety, alkyl side chain length, hydrophobicity, p*K*a value, and cell surface absorptivity are crucial aspects for antimicrobial activity [17]. In this sense, Marek et al. synthesized derivatives of pyridinium-4-aldoxime with alkyl chains of different lengths (C8 to C20) [18]. They found that the C14 and C16 derivatives were the most effective and less toxic than commercial QAS and proposed them as new potential antimicrobial candidates. In addition to their potent antibacterial potential, derivatives of pyridine oximes have also shown strong antiviral activity against *influenza B-Mass* and HIV-1 viruses [19], and some of them have been discovered as potential antidotes for organophosphate poisoning [20,21,22] because the oxime group upon deprotonation leads to a strong nucleophile that acts as a cleaver of esters and amides [23].

Inspired by previous studies [18,24], in this work we extended the structure-activity investigation by studying the effect of different aryl and alkyl substituents on the biological and physical properties of pyridinium-4-aldoxime. The biological potential of the synthesized compounds was determined by measuring the minimum inhibitory concentrations (MIC) against representative Gram-positive and Gram-negative bacteria and by determining the number of local lesions in plants infected with *Tobacco mosaic virus*. Due to the potential application of the identified candidates as antibacterial and antiviral agents, cytotoxicity was determined on healthy human cell lines, namely HaCaT and RPE1. For all compounds, the topological polar surface area and *cLogP* values were calculated to estimate the effects of the oxime group and other substituents on the physical properties of the pyridine backbone. In addition, the membranolytic mechanism of action was investigated using atomic force and inverted optical fluorescence microscopies. Using molecular dynamics (MD) simulations, we were able to predict the stability of the QacR dimer in complex with the candidate compound, which provides an explanation for the decreased antibacterial activity of the quaternary pyridinium-4-aldoxime salts.

## 2. Results and Discussion

### 2.1. Chemistry

**Scheme 1:**
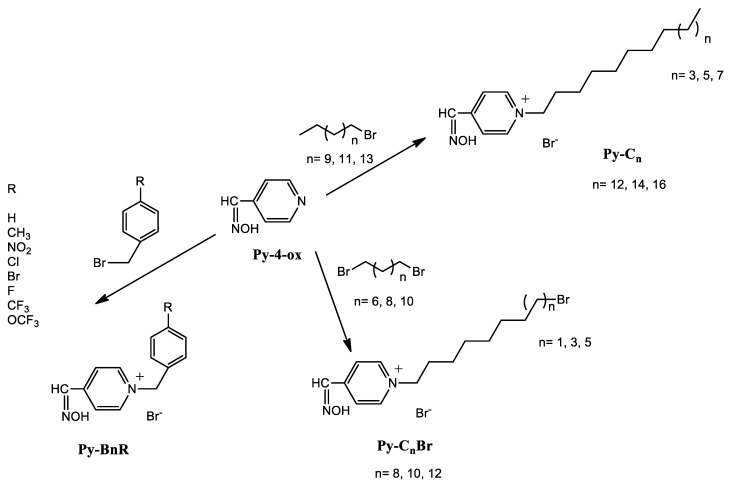
Preparation of aryl/alkyl quaternary pyridinium-4-aldoxime salts.

The preparation of monoquaternary oxime salts is a simple one-step reaction of pyridine-4-aldoxime with aryl or alkyl bromides/dibromides (Scheme 1). Nevertheless, repeated crystallization was necessary to obtain the required purity of the compounds. We must point out that some of the compounds have been previously synthesized [18,22,24]. All quaternization reactions were carried out at room temperature in acetone and the reaction products were obtained in very good yields. Alkylated QAS, more specifically, **Py-C_12_**, **Py-C_14_** and **Py-C_16,_** were obtained in better yields (*η* = 79–82%) than reported by Marek et al. who used reflux with two different solvents, acetonitrile and ethanol (*η* = 55–87% and *η* = 70–75%) [18]. Our reaction conditions using a less polar solvent at room temperature are also supported by the fact that we obtained alkylated QAS with terminal bromine atom in high yields (*η* = 60–73%).

### 2.2. In Vitro Evaluation of Biological Activity

#### 2.2.1. Antibacterial Activity

The antibacterial activity of all compounds was evaluated by determining the minimum inhibitory concentration (MIC), which is defined as the lowest concentration of the compound at which no visible bacterial growth is detected. The compounds were tested against representatives of Gram-positive and Gram-negative strains, including the ESKAPE bacterium *Staphylococcus aureus* ATCC25923 and the clinical isolate of methicillin-resistant *S. aureus* (MRSA), which are known for their pathogenicity [3].

As can be seen in Table 1 and Table 2, all pyridine-4-aldoxime QAS have much better antibacterial potential than the precursor pyridine-4-aldoxime, but higher MIC than the standard CPC. The MIC values of CPC against representative Gram-positive bacteria and *Escherichia coli* are in the μM range, in contrast to our QAS whose MICs are ranging from μM to mM values. In contrast to our previous experience with quaternization of other heterocycles [25,26], here we obtained poorly active QAS regardless of the type of substituent used for quaternization.

In this series, slightly better activities were observed for alkylated pyridinium-4-aldoxime QAS, although these activities were still in the mM range. In general, we can conclude that the alkylated QAS have better MIC for Gram-positive than for Gram-negative bacteria, which is in agreement with previous studies that also found better activity of QAS against Gram-positive strains [3]. This is mainly attributed to the different membrane structure and composition of these two types of bacteria, as Gram-negative bacteria have a double membrane that is more difficult to penetrate. We have also confirmed that chain length affects MIC, such that longer alkyl chain derivatives generally have a lower MIC [3]. Studies have shown that antibacterial activity depends mainly on the hydrophilic-hydrophobic balance of the amphiphilic compounds [27] and not solely on the length of the chain, since derivatives with longer chains have poor solubility [28]. Moreover, when investigating the antibacterial activity of pyridine-4-aldoxime QAS, Marek et al. reported the lowest MIC values for derivatives with C_12_, C_14_, and C_16_ atoms in the chain, which is in agreement with our observation [18]. Therefore, these compounds were considered as antimicrobial candidates.

In our study, **Py-C_14_** showed consistently better MIC for the series of Gram-positive strains (Table 2). Mereghetti et al. showed that of 97 isolates of *L. monocytogenes*, 17 exhibited resistance to QAS at high concentrations (MIC up to 18 mg/L) [29]. In our study, the alkylated pyridinium-4-aldoxime QAS showed promising antibacterial potential against *L. monocytogenes* ATCC7644 with the lowest MIC of 0.31 mg/mL for **Py-C_14_**. The derivative with an alkyl chain of 16 carbon atoms, **Py-C_16_**, showed the lowest MIC, but these values were limited to *S. aureus* ATCC25923 (0.09 mM), MRSA, and *Bacillus cereus* strains (0.37 mM). We must note that **Py-C_16_** has the same MIC against Gram-negative *Escherichia coli* as for Gram-positive *B. cereus*, which is consistent with the observation that QAS containing C14-C16 have the best antibacterial activity against Gram-negative strains [30]. When antibacterial efficacy was compared with standards, **Py-C_16_** was found to have 28-fold and 10-fold higher MIC for CPC and cefotaxime against *S. aureus* ATCC25923, respectively. However, MRSA had comparable MIC values for **Py-C_16_** and cefotaxime (160 and >119.4 μg/mL, respectively), leading us to conclude that this bacterial strain may use the same efflux pump system to drive bactericidal agents out of the cell. Interestingly, it has been reported that there are examples of efflux pumps that export both QAS and other antimicrobial agents. It has been shown that *qacA/B* and a gene conferring resistance to β-lactams, such as cefotaxime, are both found on large plasmids in various Staphylococcus species [31].

Compounds containing a terminal bromine atom (**Py-C_8_Br**, **Py-C_10_Br** and **Py-C_12_Br**) in an alkyl chain showed a gradual improvement in MIC values with the higher number of carbons in the chain. Nevertheless, these values were generally poor, suggesting that a large halogen atom at the end of the alkyl chain negatively affects the biological activity of QAS.

Interestingly, arylated QAS have better potential against Gram-negative strains, which may be due to the different mode of action. QAS with substituted benzyl ring showed better antibacterial potential than **Py-Bn** alone, which is probably due to the preferential substitution at *para* position. However, the best activity in this series was observed for **Py-BnCl**, **Py-BnF**, **Py-BnBr**, and **Py-BnOCF_3_** against *E. coli* with MIC values ranging from 0.43 to 2.02 mM. Similarly, **Py-BnCH_3_** and **Py-BnBr** showed MIC between 1.69 and 2.05 mM against *Salmonela enterica*. The observed potential against Gram-negative strains, especially *E. coli*, could be explained by the size of the *para* substituent or by the specific unknown mechanism of action.

#### 2.2.2. Antiviral Activity

Recent data have shown that QAS have good antiviral potential, which is why these compounds were used extensively during the COVID-19 pandemic [32]. Coronaviruses have a lipophilic membrane that is easily destroyed by the application of topical antiseptics or disinfectants [6]. Numerous plant viruses are important pathogens for agricultural crops, and new antiviral agents are welcome for economic and environmental reasons. Therefore, one of our goals was to investigate whether our new QAS have antiphytoviral potential. Tobacco mosaic virus (TMV) occupies a unique place in the history of virology and remains one of the most important pathogens of agricultural crops infecting over 200 species of herbaceous and, to a lesser extent, woody plants. The viral disease has the greatest impact on vegetables, where it can reduce yield and significantly affect quality. Extensive research has been conducted to control TMV. The most common methods include biological and chemical control. Natural products such as essential oils, flavonoids, polyphenols, and organic, alcoholic, and aqueous extracts from plants and other organisms, such as fungal metabolites, have been tested against plant diseases caused by viruses and other phytopathogens [33,34,35,36]. It is undisputed that chemical control methods continue to play an important role in disease control because of their ease of use and economic advantages. As a result, the development of efficient, environmentally friendly antiviral agents by chemical synthesis has become the core area of research to eradicate TMV and/or prevent TMV attacks. All of this has encouraged us to conduct antiviral studies that will increase our knowledge of the biological effects and potential applications of QAS.

**Figure 1:**
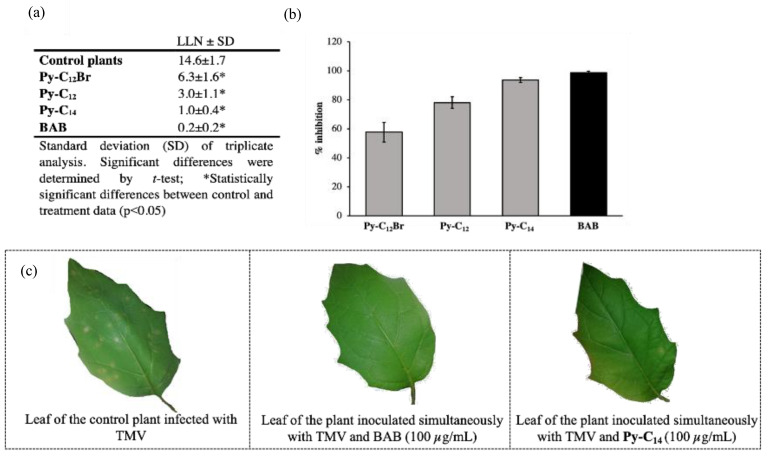
(**a**) Number of local lesions (LLN) on leaves of treated and control *Datura stramonium* plants. Treated plants were simultaneously inoculated with *Tobacco mosaic virus* (TMV) and tested compounds. (**b**) Percentage of inhibition of LLN for the tested QAS and BAB. Error bars show standard deviation of triplicate analyses. (**c**) Local symptoms of TMV infection on leaves of control plants and plants inoculated with TMV and BAB or TMV and **Py-C_14_**.

The results of the efficacy of selected alkylated pyridinium-4-aldoxime QAS, namely **Py-C_12_ Br**, **Py-C_12_**, **Py-C_14_**, and BAB against TMV infection on local host plants are shown in Figure 1. The results show that simultaneous inoculation of the tested compounds and TMV significantly reduced the number of local lesions on host plant leaves (Figure 1a). Plants treated with BAB as a control substance developed almost no infection symptoms (Figure 1c), and the percentage of inhibition of local lesions was 98.7% (Figure 1b). Among the tested series of alkylated QAS, **Py-C_14_** reduced the number of local lesions most efficiently with a percent inhibition of 93.7% (Figure 1b). This is a very promising antiviral activity that opens a new field of research for these compounds. In the same series, **Py-C_12_Br** and **Py-C_12_** had slightly lower activity, but the inhibition rate was still worth mentioning with a promising 57.7 and 78.1%, respectively. The results show that simultaneous inoculation of the tested compounds and TMV significantly reduced the number of local lesions on plant leaves (Figure 1a). The control BAB showed the highest potential to suppress the symptoms of virus infection with the lowest number of local lesions (LLN 0.2), i.e., a percentage inhibition of 98.7% compared to control plants. Among the tested series of alkylated QAS, **Py-C_14_** had 5-fold higher LLN than the control and showed almost maximum inhibition of 93.7%. In the same series, **Py-C_12_Br** and **Py-C_12_** had lower antiviral activity but inhibition greater than 50% (57.7 and 78.1%, respectively).

Thus, we can conclude that **Py-C_14_** is a new potent antiviral candidate in the series of alkylated QAS, which has similar activity to the commercial standard. Although the antibacterial and antiviral potentials are not comparable at first glance, an increasing trend in bioactivity can clearly be observed for the **Py-C_12_Br**, **Py-C_12_**, and **Py-C_14_** sequence. Despite the lower antibacterial activity, we show here that the alkylated pyridine-4-aldoxime salts have strong antiviral activity comparable to standard. This was expected since viruses lack the *qacR*/*qacA* resistance pathway typical of bacteria.

#### 2.2.3. Cytotoxic Activity

**Py-C_16_** has the best antibacterial potential and shows versatile activity against Gram-positive and Gram-negative strains, while **Py-C_14_** has the best activity against *Tobacco mosaic virus* (TMV). Therefore, these two candidates were tested for their cytotoxicity. Keratinocytes are the primary cell type found in the outermost layer of the skin. Therefore, HaCaT cells were used as a model for human skin, and RPE1 are retinal pigment epithelial cells, thus a model for epithelial cell type. These healthy human cell lines were used to test the cytotoxicity of standard CPC, precursor, **Py-4-ox**, **Py-C_14_**, and **Py-C_16_**.

**Figure 2:**
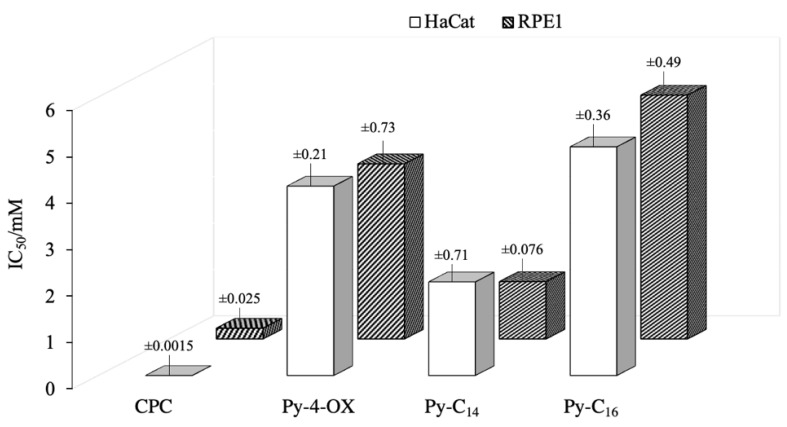
Cytotoxicity of **CPC**, **Py-4-ox**, **Py-C_14_**, and **Py-C_16_** expressed as a concentration (IC_50_/mM) of the compounds at which 50% inhibition of cell growth is observed. Experiments were performed in triplicate and results are given as means of three independent experiments, with standard deviations presented as error bars. The experiments were performed with HaCaT and RPE1 cells.

Figure 2 shows the cytotoxicity results in the form of bars representing the concentration of the compound at which 50% inhibition of cell growth is observed. The standard CPC was the most toxic and inhibited cell growth at the lowest concentrations (below 0.5 mM). The precursor **Py-4-ox**, and the candidates **Py-C_14_** and **Py-C_16_** were moderately toxic to both cell lines, with **Py-C_16_** being the least toxic. This is interesting considering that **Py-C_16_** is a structural analogue of CPC, differing only in the presence of the oxime group. Most importantly, the IC_50_ values for the identified candidates were 10- to 50-fold higher than the concentrations at which antimicrobial activity was observed. Therefore, results suggest that these new antimicrobial candidates could be considered as potentially non-toxic and safe QAS.

### 2.3. Hydrophobicity and Electron Density Distribution of Synthesized QAS

In this study, the prediction of hydrophobicity for all compounds was generated using the SwisADME online tool. This tool provides an average cLog*P* value from iLog*P*, xLog*P*, wLog*P*, mLog*P*, and silicos-it [37]. A higher cLog*P* value indicates a stronger distribution of the compound in the lipid phase, which could be a good indicator of how compounds behave in complex biological systems.

The calculated cLog*P* values are listed in Table 3. All benzylated salts of the pyridine-4-aldoxime backbone have very low cLog*P* values, indicating that these compounds are almost evenly distributed between the lipid and aqueous phases. Given their low cLog*P* values, it is reasonable to assume that they are less susceptible to membrane penetration. On the other hand, the cLog*P* values of the alkylated pyridine-4-aldoxime derivatives are much higher, suggesting that they are much more hydrophobic and, therefore, most likely to interact with the membrane. Our cLog*P* data for **Py-C_12_**, **Py-C_14_**, and **Py-C_16_** are consistent with those of Marek et al. although the values are different due to the effect of the bromide counterion [18]. It can be seen that these values are similar or close to those of CPC and BAB for alkylated derivatives. We must note that compounds with terminal bromine atom have high cLog*P* values like the standard CPC and BAB, but have low antimicrobial potential, possibly due to the strong steric hindrance caused by the large halogen atom at the end of the chain.

**Figure 3:**
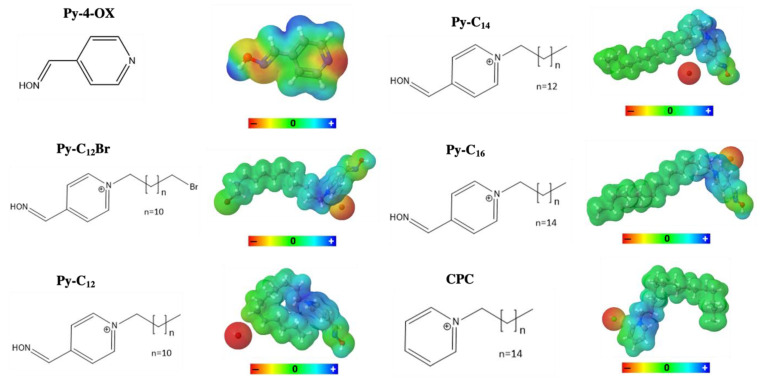
Representative structures of pyridinium-4-aldoxime and its quaternary salts with visualized topological polar surface areas (TPSA).

In addition, calculation of the topological polar surface area (TPSA) (Table 3) shows that all synthesized derivatives have higher polar surface area values than structurally similar CPC, suggesting that the addition of oxime group and/or strong electron withdrawing and polar groups, e.g., NO_2_ or OCF_3_, negatively affects the lipophilicity of the structures. This is clearly seen in the structure of the pyridine-4-aldoxime backbone (Figure 3), which has a polar surface over the oxime group and the nitrogen atom in the pyridine ring. Moreover, a comparison of **Py-C_16_** and CPC, which are both structurally similar and have similar cLog*P* values, shows a large difference in TPSA, which can be explained by the influence of the polar oxime groups on the synthesized QAS. Nevertheless, this compound shows the best biological activity. For illustration, the TPSA of the representative structures is shown in Figure 3.

### 2.4. Atomic Force Microscopy (AFM)

Optical and atomic force microscopy of the immobilized untreated and treated cells was performed to visualize the effect of **Py-C_12_** on the bacterial membrane. For these experiments we used *Escherichia coli* DH5α cells because these cells allowed solid immobilization with persevered cell viability.

**Figure 4:**
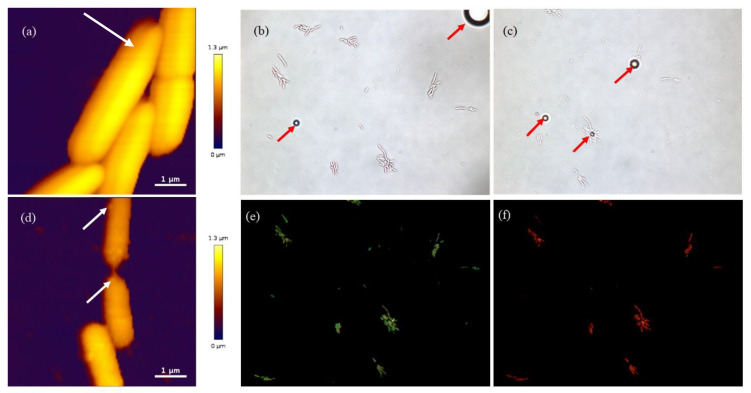
Atomic force and optical microscopy data of untreated and treated bacterial cells: (**a**) Height AFM image of untreated *Escherichia coli* DH5α cells, (**b**) bright-field image of cell groups taken at the very beginning of treatment—the arrows indicate possible vesicles, (**c**) bright-field image of the exact same sample area as in (**b**) taken after 3 h of cell treatment and vigorous rinsing with the Muller-Hinton broth—the arrows indicate possible vesicles, (**d**) height AFM image of bacterial cells—the arrows indicate possible micelles attached at the cell surface or membrane blebbing, (**e**) SYTO 9 fluorescence image of the exact same sample area as in (**b**,**c**), acquired after 3 h of treatment, (**f**) Propidium iodide fluorescence image of the exact same area as in (**e**), acquired after 3 h of treatment.

Prior to the measurements, we determined the minimum inhibitory concentration of **Py-C_12_** against *E. coli* DH5α and found it to be equivalent to that of *E. coli* ATTC 25922. The height AFM image in Figure 4a shows the untreated characteristically rod-shaped *E. coli* DH5α cells with smooth cell surface in an ongoing cell division process (white arrow). After making sure that the cells were properly immobilized and proliferating (a few new generations of cells were observed via optical microscopy), we started the treatment. To accelerate the process of time-dependent membrane damage, the cells were treated with the 4xMIC of **Py-C_12_** for three hours after which the sample was rinsed with the fresh growth medium. Bright-field images in Figure 4b,c show an unchanged number of bacterial cells at the start and immediately after the treatment, demonstrating the bacteriostatic effect of **Py-C_12_**. Interestingly, the unidentified spherical structures (red arrows) seen in these images could be from the highly aggregated **Py-C_12_**, which leads to the formation of vesicles at the concentration used (4xMIC).

These formations were stable during the 3-hour treatment and after rinsing of the cells with the growth medium (Figure 4c).

The height AFM image (Figure 4d) of the 3-hours treated cells at 4 × MIC shows an altered cell morphology with a corrugated and/or disrupted cell surface, but without massive lysis since the cells have preserved their characteristic rod shape. A similar effect was previously observed when *E. coli* cells were treated with newly synthesized quaternary *N*-benzylimidazole salts [26]. Moreover, the false color ruler, which reveals the height of the structures in the image, indicates the reduction in height of treated cells when compared to the untreated cells. Furthermore, white arrows in Figure 4d point to bulges on the cell surface that could be signs of either membrane blebbing and/or micelle attachment.

To estimate the percentage of cells whose membrane had been permeabilized by the treatment, the same group of cells was stained simultaneously with two fluorescent nucleic dyes, SYTO9 and propidium iodide (PI). Figure 4e,f show the SYTO9 staining of all cells and the PI staining of the permeabilized cells. When applied together, PI tends to displace SYTO9 from nucleic acid and the cells fluorescence in red [38]. However, there is some overlap in the SYTO9 and the PI signal, which results with different shades of green as seen in Figure 4e (the permeabilized cells tend to be green-orange in the SYTO9 signal, while the preserved cells tend to florescent bright green). Images such as Figure 4e,f allowed us to calculate the percentage of permeabilized cells—87% of the 344 cells were identified as permeabilized.

### 2.5. The Effect of Py-C_14_ Binding to the Transcriptional Factor QacR Dimer

**Figure 5:**
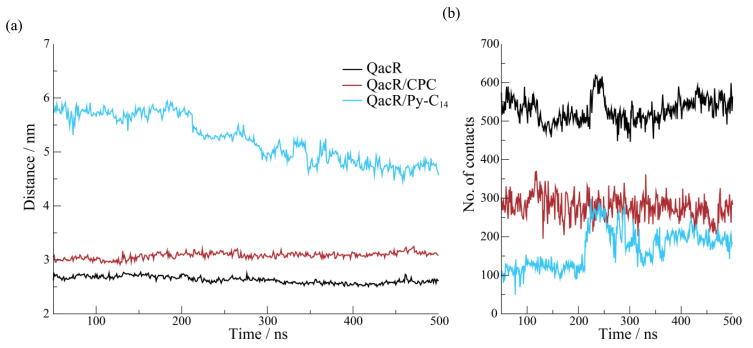
Time evolution of the distance between the centers of mass (**a**) and the number of contacts between the two monomers of the QacR protein (**b**), with the contact threshold set at 0.6 nm.

To investigate the effect of the ligand binding on the conformation of QacR, we performed MD simulations of the three systems, namely QacR dimer, QacR:CPC complex and QacR:**Py-C_14_** complex. We thereby observe rather drastic differences in the three systems (Figure 5). More precisely, one can notice that the distance between the centers of mass of the two monomeric subunits of QacR behaves in a radically different fashion depending on the presence/lack of the ligand, and also on the very nature of the ligand at hand. The average distance between the two subunits in the last 450 ns of the simulation, denoted d_m-m_, is significantly smaller in the case of both QacR (d_m-m_ ≈ 2.6 nm) and QacR:CPC (d_m-m_ ≈ 3.1 nm) systems compared to QacR:**Py-C_14_** (d_m-m_ ≈ 5.1 nm) system, see Figure 5a.

Taking only this finding into consideration, one could be prone to hypothesize the following—while the dimer remains stable in the cases of QacR and QacR:CPC systems, **Py-C_14_** ligand has a disruptive effect on the protein, causing it to dissociate into individual subunits, i.e., into two monomers. Specifically, inspection of the contacts between the two monomeric subunits reveals that QacR dimer, lacking any ligand, possesses by far the largest number of contacts between its monomeric subunits on average (≈550 contacts), compared to either QacR:CPC (≈290 contacts) or QacR:**Py-C_14_** (≈200 contacts). We thus observe the following relation: QacR:**Py-C_14_** >> QacR:CPC > QacR (Figure 5a) regarding the distance between the centers of mass of the two monomeric subunits, while QacR >> QacR:CPC > QacR:**Py-C_14_** (Figure 5b) regarding the number of contacts. It is to be noted that the inversely proportional behavior of the two trends is fully expected, as the smaller average distance indeed implies a larger number of contacts. However, such a similar number of contacts between the two monomers in the cases of QacR:CPC and QacR:**Py-C_14_** is quantitatively rather unexpected.

**Figure 6:**
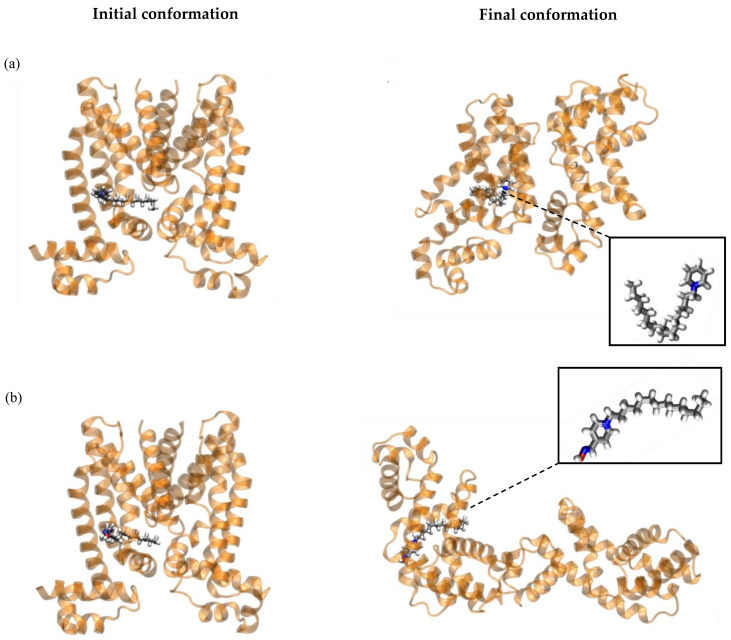
Dimer conformations representing the state of QacR during MD simulations for: (**a**) QacR:CPC at 200 ns (right; inset: the CPC ligand enlarged), (**b**) QacR:**Py-C_14_** at the beginning (starting snapshot, left) and during the last 200 ns (right; inset: **Py-C_14_** ligand enlarged). Protein is given in orange (new cartoon representation), with the ligands shown in the licorice.

In this respect, we further examined conformational behavior of the QacR protein in detail, finding that dimer reorganizes when either of the investigated ligands is present (compare right panel to the left panel of Figure 6). Evidently, binding of **Py-C_14_** first induces the dimer disruption and then reintegration of only a portion of the interactions. While both ligands have a pronounced effect on QacR, the outcome with respect to dimer *organization* is qualitatively different in the case of CPC and **Py-C_14_** (compare Figure 6b to Figure 6a). In this regard, if we imagine a monomer as a cylinder, the interactions between the monomers in the dimer could be perceived along their cylinder jackets (Figure 6, left panel). While this kind of interaction is preserved in the case of CPC ligand (Figure 6a, right), the dimer containing **Py-C_14_** shows a different arrangement, with the base-to-base interactions between the monomers being predominant (Figure 6b, right).

We also analyzed the conformational behavior of the two ligands and the specific interactions the ligands form with QacR. We thus find that rather similar initial conformations of both ligands with the aliphatic tails approximately straight (“open” states of the ligands, Figure 6, left panel), become different in the post-equilibrium stage of MD simulations. More precisely, **Py-C_14_** retains the “open” state conformation, while CPC adopts the “closed” conformation, as seen from insets of Figure 6b and Figure 6a, respectively. Finally, we also analyzed the specific interactions between the two ligands and amino acid residues belonging to the QacR monomers to which they are anchored, focusing primarily on the hydrogen bond (HB) interactions. HB is considered to have formed if the X-H···Y bond is shorter than 0.35 nm and if the angle closed with the three atoms defining HB is smaller than 30°. Due to the fact that CPC possesses only one HB acceptor (nitrogen atom of the pyridinium moiety), its ability to form HBs with the amino acids of QacR is significantly reduced compared to **Py-C_14_**, which due to the presence of the oxime group, additionally forms HBs with six amino acid residues lining the ligand binding pocket (Table 4). In light of this, it is not surprising that CPC forms no HBs during the entire course of the simulation. However, it does readily form *π-π*-interactions via its pyridinium moiety with the aromatic sidechain of Trp60, as well as favorable electrostatic interactions with Gln95 and Thr87.

On the other hand, **Py-C_14_** forms 0.3 ± 0.5 HBs on average during its respective MD simulation, with the HBs being most readily formed with the sidechains of Glu67 and Gln63 through -OH moiety of **Py-C_14_**, accounting for 73% of all HBs this ligand forms with QacR (Table 4). However, it is worth noting that, regardless of the presence/lack of the HB interactions in the case of **Py-C_14_** and CPC, respectively, both ligands remain bound to QacR for the entire 500 ns of the MD simulations.

Overall, one can conclude that binding of both ligands strongly affects the initial conformation of the QacR dimer. However, binding of **Py-C_14_**, as compared to CPC, additionally initiates the dimer dissociation and subsequent reintegration of base-to-base contacts between the monomers.

## 3. Material and Methods

### 3.1. Synthesis

The synthesis of the compounds Py-Bn, Py-BnCH_3_, Py-BnNO_2_, Py-BnCl, Py-BnBr, Py-C_12_, Py-C_14_, and Py-C_16_ with associated NMR spectra was described in [9,10,11]. However, for the purpose of this investigation, all these and new monoquaternary oximes were prepared by adding the appropriate reagents for quaternization: 4-benzyl bromide, 4-methylbenzyl bromide, 4-nitrobenzyl bromide, 4-chlorobenzylbromide, 4-bromobenzyl bromide, 4-fluorobenzyl bromide, 4-(trifluoromethyl)benzyl bromide, 4-methoxybenzyl bromide, 1, 8-dibromooctane, 1, 10- dibromodecane, 1, 12-dibromododecane, 1-bromododecane, 1-bromotetradecane, and 1-bromohexadecane; in equimolar amounts to the solution of 4-hydroxyiminomethylpyridine in dry acetone at room temperature. The reaction mixture was kept in the dark without stirring for 2–3 days to obtain a solid product. The excess of the acetone was removed under reduced pressure and the white crystals were washed several times with dry diethyl ether. The quaternary compounds were obtained as white crystals in good yields. All synthesized compounds were identified by IR and NMR spectroscopies.

Pyridinium-4-aldoxime and all reagents for quaternization were commercially available (Alfa Aesar) and were used without further purification. The progress of quite simple one-step reaction was monitored by thin layer chromatography using DC-Alufolien Aluminiumoxide 60 F_254_ plates (Merck) with 5:1 and 9:1 chloroform/methanol as eluent. Spots were detected by UV light and by reversible absorption of iodine. Repeated crystallization was necessary to achieve the required purity of the compounds. Melting points were determined in open capillaries using a Büchi B-540 instrument and are uncorrected. Elemental analyses were performed using a PerkinElmer PE 2400 Series II CHNS/O Analyzer. FTIR spectra were recorded using a PerkinElmer FTIR 1725 X spectrometer. All samples were prepared by mixing FTIR-grade KBr (Sigma-Aldrich) with 1% (*w*/*w*) salt and grinding to a fine powder. Spectra were recorded over the range 400–4000 cm^−^^1^ without baseline corrections. ^1^H and ^13^C NMR spectra were recorded in DMSO-*d*_6_ solutions using a Bruker Avance III HD 400 MHz/54 mm Ascend spectrometer (400 MHz) at room temperature. Chemical shifts are reported as *δ* values in ppm using TMS as the internal standard. Abbreviations for the data reported are: *s*, singlet; *d*, doublet; *t*, triplet; *m*, multiplet. The coupling constants (*J*) are given in Hz.

N-p*-fluorobenzyl-4-hydroxyiminomethylpyridinium bromide* (**Py-BnF**). Yield: 89%; mp: 181–183 °C; IR (KBr) *ῦ*/cm^−1^: 3313, 3075, 2924, 1666, 1466, 1220, 1008, 775. ^1^H-NMR (400 MHz, DMSO-*d*_6_): *δ*/ppm 12.85 (s, 1H, OH); 9.21 (d, 2H, *J* = 6.9 Hz, H2 and H6 Py); 8.44 (s, 1H, CH=N); 8.26 (d, 2H, *J* = 6.9 Hz, H3 and H5 Py); 7.64–7.71 (m, 2H, ArH); 7.34–7.26 (m, 2H, ArH); 5.88 (s, 2H, NCH_2_). ^13^C-NMR (100 MHz, DMSO-*d*_6_): *δ*/ppm 163.5 (q, *J_C-F_* = 246 Hz), 169.3, 145.4, 132.0, 131.0, 124.9, 116.6 (d, *J_C-F_* = 22 Hz), 62.2. Anal. Calcd. for C_13_H_12_BrFN_2_O: 50.18% C; 3.89% H; 9.00% N. Found: 50.14% C; 3.88% H; 8.97% N.

N-*(*p*-(trifluoromethyl))benzyl-4-hydroxyiminomethylpyridinium bromide* (**Py-BnCF_3_**). Yield: 97%; mp: 186–187 °C; IR (KBr) *ῦ*/cm^−1^: 3423, 3108, 2990, 1655, 1428, 1288, 1001, 782. ^1^H-NMR (400 MHz, DMSO-*d*_6_): *δ*/ppm 12.89 (s, 1H, OH); 9.22 (d, 2H, *J* = 6.7 Hz, H2 and H6 Py); 8.45 (s, 1H, CH=N); 8.29 (d, 2H, *J* = 6.7 Hz, H3 and H5 Py); 7.83 (d, 2H, *J* = 8.5 Hz, ArH); 7.76 (d, 2H, *J* = 8.5 Hz, ArH); 6.00 (s, 2H, NCH_2_). ^13^C-NMR (100 MHz, DMSO-*d*_6_): *δ*/ppm 150.6, 149.5, 145.6, 139.2, 130.1, 124.9, 124.4 (q, *J_C-F_* = 273 Hz), 62.3. Anal. Calcd. for C_14_H_12_BrF_3_N_2_O: 46.56% C; 3.35% H; 7.76% N. Found: 46.49% C; 3.35% H; 7.77% N.

N-*(*p*-trifluoromethoxy)benzyl-4-hydroxyiminomethylpyridinium bromide* (**Py-BnOCF_3_**). Yield: 61%; mp: 175–176 °C; IR (KBr) *ῦ*/cm^−1^: 3311, 3112, 2995, 1644, 1458, 1311, 1004, 765. ^1^H-NMR (400 MHz, DMSO-*d*_6_): *δ*/ppm 12.88 (s, 1H, OH); 9.22 (d, 2H, *J* = 6.7 Hz, H2 and H6 Py); 8.46 (s, 1H, CH=N); 8.28 (d, 2H, *J* = 6.7 Hz, H3 and H5 Py); 7.73 (d, 2H, *J* = 8.5 Hz, ArH); 7.47 (d, 2H, *J* = 8.5 Hz, ArH); 5.94 (s, 2H, NCH_2_). ^13^C-NMR (75 MHz, DMSO-*d*_6_): *δ*/ppm 149.4, 147.1, 145.6, 134.1, 124.9, 122.2, 121.3, 120.4 (q, *J_C-F_* = 257 Hz), 62.1. Anal. Calcd. for C_14_H_12_BrF_3_N_2_O_2_: 44.58% C; 3.21% H; 7.43% N. Found: 44.68% C; 3.20% H; 7.44% N.

N*-(8-bromooctyl)-4-hydroxyiminomethylpyridinium bromide* (**Py-C_8_Br**). Yield: 60%; mp: 100–101 °C; IR (KBr) *ῦ*/cm^−1^: 3410, 3208, 2713, 1602, 1410, 999, 538. ^1^H-NMR (400 MHz, DMSO-*d*_6_): *δ*/ppm 12.82 (s, 1H, OH); 9.10 (d, 2H, *J* = 6.7 Hz, H2 and H6 Py); 8.61 (s, 1H, CH=N); 8.25 (d, 2H, *J* = 6.8 Hz, H3 and H5 Py); 4.59 (t, *J* = 7.4 Hz, 2H, NCH_2_); 1.91–1.71 (m, 2H, CH_2_); 1.38–1.24 (m, 12H, CH_2_). ^13^C-NMR (100 MHz, DMSO-*d*_6_): *δ*/ppm 149.3, 147.7, 145.9, 123.4, 59.6, 34.6, 31.5; 29.9, 27.6, 24.7. Anal. Calcd. for C_14_H_22_Br_2_N_2_O: 42.66% C; 5.63% H; 7.11% N. Found: 42.78% C; 5.61% H; 7.13% N.

N*-(10-bromodecyl)-4-hydroxyiminomethylpyridinium bromide* (**Py-C_10_Br**). Yield: 71%; mp: 103–105 °C; IR (KBr) *ῦ*/cm^−1^: 3400, 3188, 2718, 1641, 1438, 999, 547. ^1^H-NMR (400 MHz, DMSO-*d*_6_): *δ*/ppm 12.82 (s, 1H, OH); 9.08 (d, 2H, *J* = 6.8 Hz, H2 and H6 Py); 8.60 (s, 1H, CH=N); 8.25 (d, 2H, *J* = 6.8 Hz, H3 and H5 Py); 4.61 (t, *J* = 7.3 Hz, 2H, NCH_2_); 1.92–1.71 (m, 2H, CH_2_); 1.38–1.23 (m, 16H, CH_2_). ^13^C-NMR (100 MHz, DMSO-*d*_6_): *δ*/ppm 150.6, 148.8, 145.6, 124.5, 60.67, 35.7, 32.7; 31.1, 29.2, 27.6, 25.8. Anal. Calcd. for C_16_H_26_Br_2_N_2_O: 45.52% C; 6.21% H; 6.64% N. Found: 45.34% C; 6.22% H; 6.63% N.

N*-(12-bromododecyl)-4-hydroxyiminomethylpyridinium bromide* (**Py-C_12_Br**). Yield: 73%; mp: 109–110 °C; IR (KBr) *ῦ*/cm^−1^: 3388, 3200, 2719, 1622, 1440, 1003, 543. ^1^H-NMR (400 MHz, DMSO-*d*_6_): *δ*/ppm 12.83 (s, 1H, OH); 9.01 (d, 2H, *J* = 6.8 Hz, H2 and H6 Py); 8.62 (s, 1H, CH=N); 8.26 (d, 2H, *J* = 6.7 Hz, H3 and H5 Py); 4.62 (t, *J* = 7.4 Hz, 2H, NCH_2_); 1.93–1.71 (m, 2H, CH_2_); 1.39–1.23 (m, 20H, CH_2_). ^13^C-NMR (100 MHz, DMSO-*d*_6_): *δ*/ppm 150.1, 148.4, 145.6, 123.5, 59.8, 35.1, 32.7, 31.4; 31.14, 29.3, 27.4, 24.9. Anal. Calcd. for C_18_H_30_Br_2_N_2_O: 48.02% C; 6.72% H; 6.22% N. Found: 47.91% C; 6.73% H; 6.21% N.

### 3.2. In Vitro Evaluation of Biological Activity

#### 3.2.1. Bacterial Strains

To evaluate antibacterial efficacy, the synthesized QAS were tested against representative Gram-positive and Gram-negative bacterial strains. All strains were acquired from Biognost (Zagreb, Croatia), except for the clinical isolate of methicillin-resistant *Staphylococcus aureus* (MRSA), which was kept as part of the culture collection in the Department of Chemistry, Faculty of Science, and the food isolate *Salmonella enterica*, which was kept in the culture collection of the University Department of Marine Studies in Split.

The collection included five Gram-positive strains, including *Staphylococcus aureus* (ATCC 25923 and a methicillin-resistant *Staphylococcus aureus* clinical strain MRSA), *Bacillus cereus* ATCC 14579, *Enterococcus faecalis* ATCC 29212, and *Listeria monocytogenes* ATCC 7644; and three Gram-negative strains: *Escherichia coli* ATCC 25922, *Pseudomonas aeruginosa* ATCC 27853 and the food isolate *Salmonella enterica*. All microbial strains were maintained at ™80 °C for long-term storage and subcultured on Mueller-Hinton (MH) agar (Biolife, Italy) prior to broth microdilution experiments. The MH plates were stored at +4 °C for no longer than one month.

#### 3.2.2. The Standard Curves for Bacterial Colony Forming Units per Milliliter (CFU/mL) versus A_600_

The standard growth curve was determined for each bacterial strain by plotting the A_600_ value with the number of colony-forming units in milliliter (CFU/mL).

The overnight bacterial culture was diluted (1:10, *v/v*) in Mueller-Hinton broth (MHB; Biolife) and incubated at 220 rpm at 37 °C until the mid-exponential growth phase was reached (A_600_ = 0.34–0.65). Then, 10-fold serial dilutions of the bacterial culture were prepared and 50 µL of each dilution was plated on Mueller-Hinton agar plates after measuring A_600_. The dilution plate on which 30–100 colonies grew was used to calculate CFU/mL. The optical density of a culture was measured using a spectrophotometer (Perkin Elmer Lambda Bio 40) and a densitometer (BioSan, DEN-1) relative to a blank sample of the medium. *Listeria monocytogenes* ATCC 7644 and *Enterococcus faecalis* ATCC 29212 were cultured on nutrient media containing 0.5% peptone, 0.3% yeast extract, 0.5% NaCl, and 1.5% agar. These two strains were cultured at 220 rpm and 35 °C.

#### 3.2.3. Broth Microdilution Assays

Antibacterial activity was evaluated using the Broth Microdilution Assay according to the Clinical and Laboratory Standard Institute’s Methods for Dilution Antimicrobial Susceptibility Test for Bacteria That Grow Aerobically; Approved Standard-Tenth Edition [39].

The stock solution of QAS (10 mg/mL) was prepared in 4% DMSO. Then, 100 µL of the tested QAS was added to the first well of the microtiter plate. Twofold serial dilutions in Muller-Hinton broth were performed over the entire plate at a concentration range of 5 mg/mL to 5 µg/mL. To each well, 50 µL of mid-exponentially grown inoculum containing 10^5^ CFU/mL in Mueller-Hinton broth was added and incubated at 37 °C for 18 hours. The minimum inhibitory concentration (MIC) was determined as the lowest concentration at which no visually detectable bacterial growth occurred in the wells.

#### 3.2.4. Virus and Plant Hosts

Leaves of *Nicotiana tabacum* L. cv. Samsun plants systemically infected with *Tobacco mosaic virus* (TMV) were ground in 0.06 mol/L phosphate buffer, pH 7.0 (1:1, *w*/*v*) to prepare virus inocula. Leaves were ground in 0.06 mol/L phosphate buffer, pH 7.0 (1:1, *w*/*v*), and centrifuged at low speed to prepare virus inoculum. Leaves of the local host *Datura stramonium* L. were dusted with silicon carbide (Sigma-Aldrich, St. Louis, MO, USA) before virus inoculation, and the inoculum was diluted with inoculation buffer to obtain 5–20 lesions per inoculated leaf. Experiments were performed when the experimental plants reached the 4–6 leaf stage.

#### 3.2.5. Antiphytoviral Activity Assay

The tested QAS (10 mg/mL) were dissolved in 4% DMSO and added to the virus inocula at a final concentration of 100 µg/mL. DMSO was added to the control virus inocula at the same concentration. The control and treated plants were then rubbed with the prepared inocula and the antiviral activity of the tested compounds was evaluated by the percentage inhibition of the number of local lesions on the leaves of the treated and control plants as described in [40]. The obtained data were analyzed in Excel (Microsoft Office) and significant differences were determined by *t*-test.

#### 3.2.6. Cytotoxicity

Cytotoxicity of selected QAS was performed using three different human cell lines (RPE1 and HaCaT) and compared with the standard cetylpyridinium chloride (CPC). Cells were grown in DMEM media in a humidified environment with 5% CO_2_ and 37 °C. To determine cytotoxicity, a twofold serial dilution of a tested compound starting at 5 mg/mL was prepared in a 96-well plate. Five thousand cells were pipetted into each well, and the cells were grown for an additional 48 h. Then, the reagent MTS (20 μL) was added to the cells according to the manufacturer’s instructions (CellTiter 96^®^ Aqueous One Solution Cell Proliferation Assay, Promega). After 3 h of incubation, the absorbance was measured at 490 nm. IC_50_ values were determined by plotting compound concentration against absorbance using GraFit6 software [41]. Measurements were performed in duplicates and results are reported as means of at least three independent experiments with standard deviation (±SD) indicated.

### 3.3. SwissADME Calculations and Visualization of Electron Density Distribution

CLogP values and topological polar surface area (TPSA) were calculated using the online tool SwissADME available at http://www.swissadme.ch (accessed on 4 March 2022). The chemical structures of the compounds were drawn using Chem Draw [42] and the generated list of SMILES (Table 5) was used to perform the corresponding calculations. The topological polar surface area (TPSA) was visualized using Jmol [43].

### 3.4. Atomic Force Microscopy

AFM measurements of immobilized cells, both untreated and treated, were performed using the Nano-wizard IV system (JPK/Bruker, Berlin, Germany) operating in quantitative imaging (QI) mode with SNL-B probes (Bruker, Billerica, MA, USA). The AFM system was integrated with an IX73 inverted fluorescence optical microscope (Olympus, Tokyo, Japan). *Escherichia coli* DH5α cells and FluoroDish Petri dishes (WPI, Sarasota, FL, USA) coated with Cell-Tak (Corning, NY, USA) solution were prepared as previously reported [44]. After preparing the coated dish, a 30 µL aliquot of the exponentially grown bacterial cells was applied and rinsed vigorously with MHB after 10 min. The final volume of the medium in the dish was 1 mL, while the temperature was always kept at 37 °C. The immobilized bacterial cells were examined with the bright-field microscope to select the AFM imaging region of choice. The cells were then grown for 2 hours under constant conditions to confirm intact cell division and elongation processes. After incubation, the cell culture was once again washed vigorously with fresh MHB, and AFM images of untreated cells were acquired. Cell treatment was initiated by adding a **Py-C_12_** stock solution to reach the final concentration equivalent to 4 × MIC. After 3 h of treatment, cells were washed again with fresh MHB and AFM images of treated cells were acquired. During the imaging of the treated and untreated cells, the set point was maintained at 0.9 nN, the extend/retract speed was between 100 and 150 µms^−^^1^ while the Z length was up to 6000 nm. The resolution of each measurement was 128 × 128 pixels. Finally, the collected AFM data were processed using JPK data processing software.

To confirm cell membrane permeation, **Py-C_12_** treatment was replaced with physiological saline (1 mL) containing 1.5 µL of each fluorescent dye—the green fluorescent SYTO 9, and the red fluorescent propidium iodide (PI), which are components of the LIVE/DEAD BacLight Bacterial Viability Kit L7012 (Invitrogen, Carlsbad, USA). Fluorescence images were taken 30 min after addition of the dyes.

### 3.5. Docking Analysis

We performed all-atom molecular dynamics (MD) simulations of QacR protein in the presence of CPC (QacR:CPC system) and **Py-C_14_** (QacR:**Py-C_14_** system) ligands, as well as in their absence (QacR system). In this respect, the starting protein structure was obtained from crystal structure with PDB code 3BTJ (dimer). The dimer (crystal water, ions, and the ligand present in the 3BTJ pdb file removed) was solvated using 40,000 water molecules (rectangular periodic boundary conditions), while chloride anions were added to each simulated system to neutralize the overall charge of the prepared simulation boxes (four chloride ions in the case of the “pure” protein, five in the cases of QacR:ligand systems). A common and consistent set of parameters was used to describe the protein and its surrounding, namely AMBER ff14SB force field [45] was used to describe the protein, with chloride anions and water molecules were represented using TIP3P water model and parameters developed by Cheatham III et al. [46], respectively. The two ligands investigated in this study were parameterized according to general AMBER force field (GAFF) [47], whereby the only missing parameters, namely partial charges of the two ligands, were calculated employing a restrained single-conformer fit to the electrostatic potential (RESP) [48]. The electrostatic potential via which partial charges were estimated was obtained using quantum mechanical calculations (HF/6-31G(d)//B3LYP/6-31G(d) level of theory). The ligands were positioned in QacR dimer on the basis of the position of the ligand (dequalinium, DEQ) found in the aforementioned crystal structure, which was performed utilizing the program Maestro from Schrödinger software suite [49].

All three prepared systems (QacR, QacR:CPC, QacR:**Py-C_14_**) were subjected to the equivalent minimization/equilibration procedure, consisting of the following steps: (a) minimization of the systems employing steepest descent algorithm (5000 steps), (b) relaxation of the systems in the duration of 10 ns at *T* = 310 K (NVT ensemble, 2 fs time step, Berendsen thermostat with time constant for temperature coupling equal to 1 ps, position restraints applied on all heavy atoms of the ligands (if present) and the protein (500 kJ mol^−1^ nm^−2^)), (c) equilibration at *T* = 310 K in the duration of 10 ns (NPT ensemble, 2 fs time step, Berendsen thermostat with time constant for temperature coupling equal to 1 ps, Berendsen barostat with *p* = 1 bar and time constant for pressure coupling equal to 5.0 ps), position restraints on all heavy atoms of the protein and ligands (200 kJ mol^−1^ nm^−2^). Upon equilibration, all three prepared system were propagated at *T* = 310 K with no applied positional restraints (free MD) in the duration of 500 ns (MD parameters of the production simulations: NPT ensemble, 2 fs time step, Nosé-Hoover thermostat with time constant for temperature coupling set to 1 ps, Parrinello-Rahman barostat with *p* = 1 bar and time constant for pressure coupling equal to 5.0 ps). All aforementioned simulations were performed taking into account periodic boundary conditions, where the particle mesh Ewald method was employed to properly account for the long-range electrostatic interactions beyond a 1.2 nm cutoff [50]. In the subsequent analysis, the first 50 ns of each individual simulation were ignored, corresponding to the equilibration period of the simulations. All MD simulations and subsequent analyses were produced using GROMACS 2020 software package [51]. The systems are visualized via VMD visualization software [52].

## 4. Conclusions

In this paper we describe synthesis and biological evaluation of quaternary ammonium salts (QAS) with aryl and alkyl substituents on pyridine-4-aldoxime. We noticed that alkyl, as opposed to aryl substituted quaternary oximes, generally show much better antibacterial potential with lowest MIC values ranging from 0.04 to 0.31 mg/mL. Moreover, the same compounds exhibit potent antiphytoviral activity against *Tobacco mosaic virus* (TMV) with **Py-C_14_** inhibiting up to 93.7% of viral load comparable to the standard compound BAB. More importantly, the identified candidates did not show toxicity toward healthy human cell lines, namely RPE1 and HaCaT, implicating that these compounds might be new potent antimicrobial agents. Furthermore, the compounds have a membranolytic mode of action, inducing an alteration of bacterial morphologies displaying corrugated and/or disrupted cell surfaces. While the presence of the oxime group unfavorably increases the topological polar surface area as compared to structurally similar CPC, the candidate compounds still exhibit potent membranolytic activity with 87% of the cells with permeabilized membrane after 3 h of treatment at 4 × MIC. The effect of new compounds on the most studied QAS resistance system involving QacR transcriptional regulator was investigated by MD simulations. We observed strikingly different QacR conformations in the presence of either ligand, CPC or **Py-C_14_**. However, the detrimental effect of **Py-C_14_** binding was most pronounced as this ligand induced QacR dimer dissociation and its re-assembly during the respective MD simulation suggesting that this class of compounds could induce a premature activation of QAS resistance system.

## Figures and Tables

**Table 1:** Antibacterial activities of benzylated derivatives of pyridinium-4-aldoxime. The minimal inhibitory concentrations (MIC) are expressed in mg mL^−1^ and mM units. MIC is the highest concentration of the compound required to suppress bacterial growth in three replicate experiments.

Bacterial Strain	Strain Origin	MIC (mg mL^−1^/mM)								
		Py-4-ox	Py-Bn	Py-BnCH_3_	Py-BnNO_2_	Py-BnCl	Py-BnF	Py-BnBr	Py-BnCF_3_	Py-BnOCF_3_
		*M*_r_ = 122.1	*M*_r_ = 293.2	*M*_r_ = 307.2	*M*_r_ = 338.2	*M*_r_ = 327.6	*M*_r_ = 311.2	*M*_r_ = 372.1	*M*_r_ = 361.2	*M*_r_ = 377.2
**Gram-positive bacteria**										
*Staphylococcus aureus*	ATCC25923	>5/>40.9	>5/>17.05	2.5/8.14	5/14.78	1.25/3.82	5/16.07	1.25/3.36	0.63/1.74	**0.31/0.82**
*Staphylococcus aureus*	Clinical/MRSA	>5/>40.9	>5/>17.05	5/16.28	>5/>14.78	2.5/7.63	>5/>16.07	1.25/3.36	1.25/3.46	1.25/3.31
*Bacillus cereus*	ATCC14579	>5/>40.9	>5/>17.05	5/16.28	>5/>14.78	5/15.26	>5/>16.07	2.5/6.72	1.25/3.46	2.5/6.63
*Enterococcus faecalis*	ATCC29212	5/40.9	>5/>17.05	2.5/8.14	>5/>14.78	5/15.26	>5/>16.07	2.5/6.72	2.5/6.92	2.5/6.63
*Listeria monocytogenes*	ATCC7644	>5/>40.9	>5/>17.05	1.25/4.07	>5/>14.78	2.5/7.63	>5/>16.07	2.5/6.72	2.5/6.92	2.5/6.63
**Gram-negative bacteria**										
*Escherichia coli*	ATCC25922	>5/>40.9	1.25/4.26	5/16.28	2.5/7.39	**0.31/0.95**	**0.63/2.02**	**0.16/0.43**	2.5/6.92	**0.31/0.82**
*Salmonella enterica*	Food isolate	5/40.9	>5/>17.05	**0.63/2.05**	5/14.78	2.5/7.63	>5/>16.07	**0.63/1.69**	1.25/3.46	2.5/6.63
*Pseudomonas aeruginosa*	ATCC27853	>5/>40.9	>5/>17.05	>5/>16.28	>5/>14.78	5/15.26	>5/>16.07	5/13.44	5/13.84	2.5/6.63

**Table 2:** Antibacterial activities of alkylated derivatives of pyridinium-4-aldoxime. The minimal inhibitory concentrations (MIC) are expressed in mg mL^−^^1^/mM and μg mL^−1^/μM. MIC is the highest concentration of the compound required to suppress bacterial growth in three replicate experiments.

Bacterial Strain	Strain Origin	MIC (mg mL^−1^/mM)						MIC (μg mL^−1^/μM)	
		Py-C_8_Br	Py-C_10_Br	Py-C_12_Br	Py-C_12_	Py-C_14_	Py-C_16_	CPC	Cefotaxime
		*M*_r_ = 394.2	*M*_r_ = 422.2	*M*_r_ = 450.3	*M*_r_ = 371.4	*M*_r_ = 399.4	*M*_r_ = 427.5	*M*_r_ = 339.9	*M*_r_ = 477.5
**Gram-positive bacteria**									
*Staphylococcus aureus*	ATCC25923	5/12.68	0.63/1.49	**0.16/0.36**	**0.08/0.22**	**0.08/0.20**	**0.04/0.09**	1.4/4.1	3.7/10
*Staphylococcus aureus*	Clinical/MRSA	2.5/6.34	0.31/0.73	**0.31/0.69**	**0.31/0.83**	**0.16/0.40**	**0.16/0.37**	2.7/10	>119.4/>250
*Bacillus cereus*	ATCC14579	2.5/6.34	0.63/1.49	0.63/1.40	1.25/3.37	1.25/3.13	**0.16/0.37**	5.3/20	3.7/10
*Enterococcus faecalis*	ATCC29212	2.5/6.34	5/11.84	1.25/2.78	0.63/1.70	**0.31/0.78**	2.5/5.85	2.7/10	29.8/60
*Listeria monocytogenes*	ATCC7644	1.25/3.17	5/11.84	1.25/2.78	0.63/1.70	**0.31/0.78**	1.25/2.92	2.7/10	3.7/10
**Gram-negative bacteria**									
*Escherichia coli*	ATCC25922	1.25/3.17	0.63/1.49	0.63/1.40	0.63/1.70	0.63/1.58	**0.16/0.37**	5.3/20	0.2/0.4
*Salmonella enterica*	Food isolate	>5/>12.68	5/11.84	2.5/5.55	0.63/1.70	2.5/6.26	5/11.70	21.2/60	0.1/0.2
*Pseudomonas aeruginosa*	ATCC27853	>5/>12.68	>5/>11.84	2.5/5.55	2.5/6.73	2.5/6.26	2.5/5.85	850/250	59.7/130

**Table 3:** Consensus partition coefficients (cLogP) and topological polar surface area (TPSA/Å^2^) of compounds.

	Compound	cLog*P*	TPSA/Å²
	Py-4-ox	0.73	45.48
**Aryl substituent**	Py-Bn	0.95	36.47
	Py-BnCH_3_	0.69	36.47
	Py-BnNO_2_	-0.62	82.29
	Py-BnCl	0.86	36.47
	Py-BnF	0.63	36.47
	Py-BnBr	0.93	36.47
	Py-BnCF_3_	1.50	36.47
	Py-BnOCF_3_	0.90	45.70
**Alkyl substituent**	Py-C_8_Br	1.76	36.47
	Py-C_10_Br	2.40	36.47
	Py-C_12_Br	3.12	36.47
	Py-C_12_	1.51	36.47
	Py-C_14_	2.07	36.47
	Py-C_16_	2.82	36.47
	CPC	3.24	3.88
	BAB	3.55	0.00

**Table 4:** Contribution of all the amino acid residues forming hydrogen-bonds (HB) with **Py-C_14_** to the overall **Py-C_14_**—protein HB count.

Amino Acid	Overall Hydrogen-Bond (HB) Count (%)
Glu67	50
Gln63	23
Leu94	12
Tyr91	9
Thr87	5
Leu80	1

**Table 5:** Compound abbreviations and list of generated smiles used for ADME calculations.

Compound	SMILES
Py-4-ox	ON=CC1=CC=NC=C1
Py-Bn	ON=CC1=CC=[N+](CC2=CC=CC=C2)C=C1.[Br-]
Py-BnCH_3_	CC(C=C1)=CC=C1C[N+]2=CC=C(C=NO)C=C2.[Br-]
Py-BnNO_2_	ON=CC1=CC=[N+](CC2=CC=C([N+]([O-])=O)C=C2)C=C1.[Br-]
Py-BnCl	ClC(C=C1)=CC=C1C[N+]2=CC=C(C=NO)C=C2.[Br-]
Py-BnF	FC(C=C1)=CC=C1C[N+]2=CC=C(C=NO)C=C2.[Br-]
Py-BnBr	BrC(C=C1)=CC=C1C[N+]2=CC=C(C=NO)C=C2.[Br-]
Py-BnCF_3_	ON=CC1=CC=[N+](CC2=CC=C(C(F)(F)F)C=C2)C=C1.[Br-]
Py-BnOCF_3_	ON=CC1=CC=[N+](CC2=CC=C(OC(F)(F)F)C=C2)C=C1.[Br-]
Py-C_8_Br	ON=CC1=CC=[N+](CCCCCCCCBr)C=C1.[Br-]
Py-C_10_Br	ON=CC1=CC=[N+](CCCCCCCCCCBr)C=C1.[Br-]
Py-C_12_Br	ON=CC1=CC=[N+](CCCCCCCCCCCCBr)C=C1.[Br-]
Py-C_12_	ON=CC1=CC=[N+](CCCCCCCCCCCC)C=C1.[Br-]
Py-C_14_	ON=CC1=CC=[N+](CCCCCCCCCCCCCC)C=C1.[Br-]
Py-C_16_	ON=CC1=CC=[N+](CCCCCCCCCCCCCCCC)C=C1.[Br-]
CPC	CCCCCCCCCCCCCCCC[N+]1=CC=CC=C1.[Cl-]
BAB	C[N+](CC1=CC=CC=C1)(C)CCCCCCCCCCCC.[Br-]

## Data Availability

Data is contained within the article.
